# Combined Adjuvant of Poly I:C Improves Antitumor Effects of CAR-T Cells

**DOI:** 10.3389/fonc.2019.00241

**Published:** 2019-04-17

**Authors:** Shengmeng Di, Min Zhou, Zeyan Pan, Ruixin Sun, Muhua Chen, Hua Jiang, Bizhi Shi, Hong Luo, Zonghai Li

**Affiliations:** ^1^State Key Laboratory of Oncogenes and Related Genes, Shanghai Cancer Institute, Renji Hospital, Shanghai Jiaotong University School of Medicine, Shanghai, China; ^2^CARsgen Therapeutics, Shanghai, China

**Keywords:** chimeric antigen receptor, poly I:C, solid tumor, MDSC, type I IFN

## Abstract

Chimeric antigen receptor modified T cells (CAR-T) therapy is an emerging immunotherapy against malignancies. However, only limited success was obtained in solid tumors. Polyinosinic-polycytidylic acid (poly I:C), ligand of TLR3, mediates innate immune and adaptive immune and shows broad antitumor effect on many types of cancer. In the present study, we combined EGFRvIII-targeted CAR-T cells with poly I:C treatment and evaluated the synergic antitumor effect *in vitro* and in immunocompetent mice bearing subcutaneous colon or orthotopic breast cancer xenografts. Poly I:C significantly promoted more IL-2 and IFN γ production as well as higher lytic activity of CAR-T cells. Upon systemic administration *in vivo*, CAR-T cells obviously suppressed tumor growth, and poly I:C significantly enhanced the suppression. Further study showed that poly I:C exerted antitumor effect dependent on type I IFNs. In addition, poly I:C decreased myeloid-derived suppressor cells (MDSC) number in peripheral blood and spleen, and attenuated the immunosuppressive activity of MDSC on proliferation and cytolytic function of CAR-T. Depletion of MDSC with anti-Gr1 Ab further increased the antitumor effect of CAR-T cells plus poly I:C treatment. In conclusion, CAR-T treatment combined with intratumoral delivery of poly I:C resulted in synergistic antitumor activity. We thus provide a rationale to translate this immunotherapeutic strategy to solid tumors.

## Introduction

Adoptive T cell immunotherapy has been demonstrated to be a new way to fight malignancies. In particular, T lymphocytes engineered to express chimeric antigen receptor (CAR) have shown great promise in treating hematological malignancies (1). CD19-targeted CAR-T cells have been approved by FDA to treat relapsed B cell acute lymphoblastic leukemia (B-ALL) and Diffuse Large B-cell lymphoma (DLBCL) (2, 3).

CAR-T therapy is also a novel approach to treat other malignant tumors. Currently, Glypian-3 (GPC3), epidermal growth factor receptor (EGFR), human epidermal growth factor receptor2 (HER2), carcinoembryonic antigen (CEA), disialoganglioside 2 (GD2), mesothelin, prostate-specific membrane antigen (PSMA), and interleukin-13Ra2 (IL13Ra2) have been tested as targets of CAR-T cells in solid tumor. However, CAR-T therapy for solid tumors has not been as efficient as those targeting hematologic malignancies (4–7).

The reasons for the limited success of CAR T cells in solid tumor are being actively investigated, and are likely multifactorial. One major reason is the immunosuppressive microenvironment that may impede the function and persistence of CAR T cells. This includes the presence of regulatory T cells, tumor-associated macrophages, myeloid-derived suppressor cells (MDSC), and cancer-associated fibroblasts, which promote higher levels of inhibitory ligands and cytokines (8). Furthermore, numerous immune checkpoint molecules, such as PD-1, CTLA-4, TIM-3, LAG-3, B7S1 (9), promote T-cell exhaustion, and dampen T-cell activation within tissues. Consequently, blockade of immune checkpoint molecules improved the antitumor activity of CAR-T cells (10). In addition, Indoleamine 2,3-dioxygenase (IDO) is an intracellular enzyme that mediates the metabolism of the essential amino acid tryptophan into immunosuppressive metabolites. Tumor IDO activity can inhibit CAR-T therapy through the action of tryptophan metabolites, while IDO inhibitor restored tumor control in a xenograft lymphoma model (11). These data suggested that targeting tumor immune-suppressive environment is potential approach for boosting the antitumor immunity of CAR-T cell.

Polyinosinic-polycytidylic acid (poly I:C), a synthetic analog of double-stranded RNA (dsRNA), is recognized by toll-like receptor 3 (TLR3), dsRNA-activated protein kinase (PKR), retinoic acid-inducible gene I protein (RIG-I) and melanoma differentiation- associated protein 5 (MDA5) (12). The application of poly I:C in tumor immunotherapy has been well investigated for several decades. Poly I:C activates the innate immune system, with subsequent regulation of adaptive immunity (13), leading to alterations in the tumor microenvironment and a striking suppression of tumor growth (14, 15). In addition, poly I:C was reported to be able to prolong CD4+ T cell survival (16), promote activated T cell proliferation (17), and reactivate tumor infiltrating CD8+ T cells (18). Moreover, studies showed that poly I:C could directly trigger cancer cells to initiate apoptosis, so that poly I:C could reduce tumor metastasis in immunodeficient mice (19). This built the rationale to combine poly I:C with CAR-T cell therapy.

In this study, we explore the potential of using poly I:C to increase the antitumor activities of CAR-T cells and the underlying mechanism.

## Materials and Methods

### Cell Lines and Medium

The murine colon carcinoma line CT26 (EGFRvIII^NEG^) was maintained in our laboratory. Human embryonic kidney cell line 293T was purchased from the American Type Culture Collection (ATCC; Manassas, VA). Both cells were grown as monolayer in DMEM culture medium (Gibco) containing 10% fetal bovine serum (FBS, Biowest) and 1% L-glutamine. Murine breast cancer cell line E0771 (EGFRvIII^NEG^, gifted by Dr. Xiang Zhang of Baylor College of Medicine) was cultured in RPMI 1,640 medium (Gibco) supplemented with 10% FBS and 10 mmol/L HEPES. All cells were routinely maintained at 37°C in a 5% CO_2_ atmosphere incubator. These cell lines were tested on a regular basis for mycoplasma and were negative.

### Murine EGFRvIII Construction

A murine homolog of the human EGFRvIII mutation was created by using cDNA sequences spanning the murine EGFR according to report (20). Briefly, the cDNA sequences cloned into pPWT vector to construct the recombinant murine EGFRvIII were as follows: base pairs 60-147, GT, and 951-3737. This cloning procedure creates a junctional peptide (LEEKKGNYVVTDH) identical to that found in human EGFRvIII. Replication-defective lentiviral vectors containing the recombinant murine EGFRvIII were then generated by 293T packaging cell lines and used to transfect CT26 and E0771 cells. Transfected cells were incubated with ch806 Ab followed with FITC labeled goat anti human IgG, then positive cells were sorted by flow cytometer.

### Proliferation Analysis

Tumor cells were plated at 10^4^ cells/well in 96-well plates with different concentrations of poly I:C (0.5 ng/mL,5 ng/mL, 50 ng/mL, 500 ng/mL, 5 μg/mL). Activated T cells were seeded in 96-well plates with poly I:C concentrations of 10 and 50 μg/mL. Cell proliferation was analyzed by CCK8 method at 24, 48, and 72 h, respectively.

### CAR Design and Generation of CAR-T Cells

The recombinant murine EGFRvIII specific CAR retrovirus was generated as follows. 806 scFv (21) was inserted in tandem with mCD8 trans-membrane, mCD28 and mCD3 ζ intracellular regions in the MSCV retroviral vector. Retroviral supernatant was generated by transient cotransfection of HEK 293T cells by PEI transfection reagent, along with pCL-Eco helper plasmid (gifted by Prof. Yongzong Liu of Shanghai Cancer Institute). Forty eight hours later, supernatant was collected and used to transduce murine splenic T lymphocyte. In short, mouse splenocytes were collected from healthy Balb/c or C57 mice, disaggregated, and passed through a 70 μm mesh filter. After lysis of red blood cells, CD3+ T lymphocytes were isolated with an EasySep™ Mouse T cell Isolation Kit (Stemcell). Purified T cells were activated with anti-CD3 and anti-C28 Ab coated dynabeads (Gibco) for 24 h and retrovirally transduced. Then cells were cultured in RPMI 1,640 media supplemented with 10% FBS (Gibco), 100 IU/mL rhIL2, 1 ng/mL rmIL-7 and 50 μmol/L β-mercaptoethanol for 3–5 days and subsequently used for experiment.

### Flow Cytometric Analysis

T cell transduction was measured using EGFP/Biotin labeled EGFRvIII protein generated in our laboratory, followed by PE-streptavidin when needed. T cell phenotypes were identified using the following antibodies: mouse CD3e (clone 145-2C11, Affymetrix eBioscience) conjugated to FITC and mouse CD8α (clone 53-6.7, Affymetrix eBioscience) conjugated to PE.

To detect MDSC from blood, spleen and tumors of mice, tumor-bearing mice were euthanized 24 h after last poly I:C treatment. Fifty microliter of peripheral blood drawn from the cheek vein was incubated with antibodies to mouse CD11b (clone M1/70, Biolegend) and Gr1 (clone RB6-805, BD bioscience) followed by red blood cell removal and then was analyzed by flow cytometry. Single cell suspensions of spleen were obtained by mechanical disruption through a 70 μm cellular sieve with a plunger, and red cells were then removed. Tumor cell suspensions were obtained by digesting dissected tumor tissues with tissue dissociation kit (Miltenyi). All antibodies were used according to manufacturer's recommendations. Live cells were gated by forward scatter/side scatter (FSC/SSC). Analysis was performed using a FACSCelesta flow cytometer (BD Biosciences) and FlowJo software (TreeStar).

### *In vitro* Cytotoxicity Assay

The activity of CAR-T cells to kill target cells *in vitro* was evaluated using lactate dehydrogenase (LDH) release assay (CytoTox 96® NonRadioactive Cytotoxicity Assay, Promega). Target cells were incubated with CAR transduced effector T cells at varying effector-to-target (E:T) ratios. After 18 h of co-incubation at 37°C, supernatant was transferred to a new microplate and the LDH released into the supernatant was assessed using a Microplate Reader. Specific cytolysis was calculated by using the formula: (Test–effector control–target control/Tmax control–target control) × 100, in which “Tmax control” was the value obtained from supernatant of target cells exposed to 1% Triton-X 100, “effector control” was the spontaneous LDH release value of CAR-T alone, and “target control” was the spontaneous LDH release value of target cells alone; background control (the value obtained from medium alone) was subtracted from each value before the calculation.

### Cytokine Release Assay

Murine CAR-T cells were tested for antigen-specific activity in cytokine release assay using tumor cells. In these experiments, effector cells were co-cultured with an equal number of target cells in complete RPMI 1,640 medium in a final volume of 0.2 ml in triplicate wells of a 96-well microplate. Culture supernatants were harvested 24 h after the initiation of co-culture and assayed for IL-2 and IFN γ by ELISA (Multisciences). Serum IFN γ level of treated tumor-bearing mice was detected by ELISA (Multisciences).

### *In vivo* Antitumor Effect of Combined Poly I:C and CAR-T Cells

All animal procedures were approved by the Shanghai Cancer Institute Committee for the Use and Care of animals and performed in accordance to the protocol. For tumor induction, 6–7 weeks old female Balb/c mice were irradiated with 3 Gy whole-body irradiation and then 3 × 10^5^ C26-EGFRvIII cells suspended in 200 μl PBS were injected s.c. into the flank of mice. To establish orthotopic breast tumors, we injected 5 × 10^5^ E0771-EGFRvIII cells into the 4th inguinal mammary fat pads of 6–7 weeks old female C57BL/6 mice. Tumor bearing mice were treated with 5 × 10^6^ CAR-T cells by tail vein injection. Mice of control groups were injected with same amount of untransduced T (UT) cells. Fifty micrograms high molecular weight poly I:C (pIC, InvivoGen) was injected intratumorally 3 and 6 days after CAR-T infusion. Mice intratumorally injected with the same volume of saline were used as control. Tumor volume was estimated using the digital caliper measurement (0.5 × length × width × width) twice a week. Mice were euthanatized when the tumor volume reached 2,000 mm^3^.

### *In vivo* Mouse IFN-β and Type I IFN Blocking

Tumor-bearing mice were injected i.t. with 50 μg poly I:C. Peripheral blood was collected from cheek veins at the indicated times. Saline was used as control. IFN-β level in sera was quantified with ELISA. To interfere type I IFN signaling, 50 μg anti-IFNAR1 mAb (clone MAR1-5A3, Bioxcell) was intratumorally injected on day 0 and 2 after poly I:C or saline treatment (22).

### Real-time PCR for CAR-T Cell Persistence

To determine the copy number of integrated CAR in genetically modified T cells, 100 ng DNA genomic DNA was extracted from the tumors of treated mice with the QIAamp DNA Mini Kit (Qiagen). DNA was amplified in triplicate with primers and TaqMan probes specific for the CAR transgenes, using the 7,500 Real Time PCR System (Applied Biosystems) in a PCR reaction (2 min for 50°C, 10 min for 95°C, followed by 45 cycles of 15 s 95°C and 1 min 60°C). To generate DNA standards, we established serial dilution of DNA plasmids encoding the specific cassette. The primers used were as follows: Primer-F: 5′- GACGTTGGGTTACCTTCTG C-3′), Primer-R: 5′- TTCCCAGGTCACGATGTAGG−3′ and probe: 5′-(FAM)-ATGGCCGCGA GACGGCACCT-(BHQ1)-3′.

### MDSC Suppression Assays

MDSCs were isolated via Ly6G magnetic selection from spleens of tumor-bearing mice 24 h after poly I:C or saline treatment. Isolated MDSCs were titrated into CAR-T-cell cultures at varying MDSC: CAR-T cell ratios and incubated overnight. For flow cytometry-based proliferation assays, mouse T cells were pre-labeled with CSFE (ThermoFisher Scientific). Anti-CD3 and anti-C28 Abs coated dynabeads were added at initial time. After 72 h, cells were collected and CFSE expression was detected by flow cytometry.

To evaluate the MDSC suppression on CAR-T lytic activity, CAR-T cells and target cells were co-cultured at an E:T ratio of 0.3:1. Isolated MDSC were added at varying MDSC: CAR-T-cell ratios. Cytotoxicity efficiency was calculated after 18 h of co-incubation.

### *In vivo* Gr1+ Cell Depletion

Tumor-bearing mice were treated with poly I:C and CAR-T cells as described above. To deplete *in vivo* MDSC, mice were intraperitoneally injected with anti-Gr1 mAb (10 mg/kg, clone RB6-8C5, BioXell) 24 h before poly I:C initially and then three times per week for 2 weeks (23). MDSC depletion was validated by flow cytometry analysis of CD11b+ Gr1+ circulating cells in peripheral blood 1 day later. Control mice were injected with IgG control mAb (10 mg/kg, clone LTF-2, BioXcell). Tumor growth was monitored twice a week. Mice were euthanatized when the tumor volume reached 2,000 mm^3^.

### Statistical Analysis

In all experiments, statistical significance was evaluated with unpaired *t*-test when comparing two groups and one-way ANOVA followed by Newman-Keuls post analysis for comparisons of 3 or more groups. Two-way ANOVA with Bonferroni post-tests was used to when comparing two groups with two different samples. The compilation of graphs and the statistical analyses were performed with Prism software (GraphPad). Data were presented as the mean values ± SEM. In all cases, *p*-values < 0.05 were considered statistically significant.

## Results

### Poly I:C Enhances the Antigen-specific Responses of CAR-T Cells

**Figure 1:**
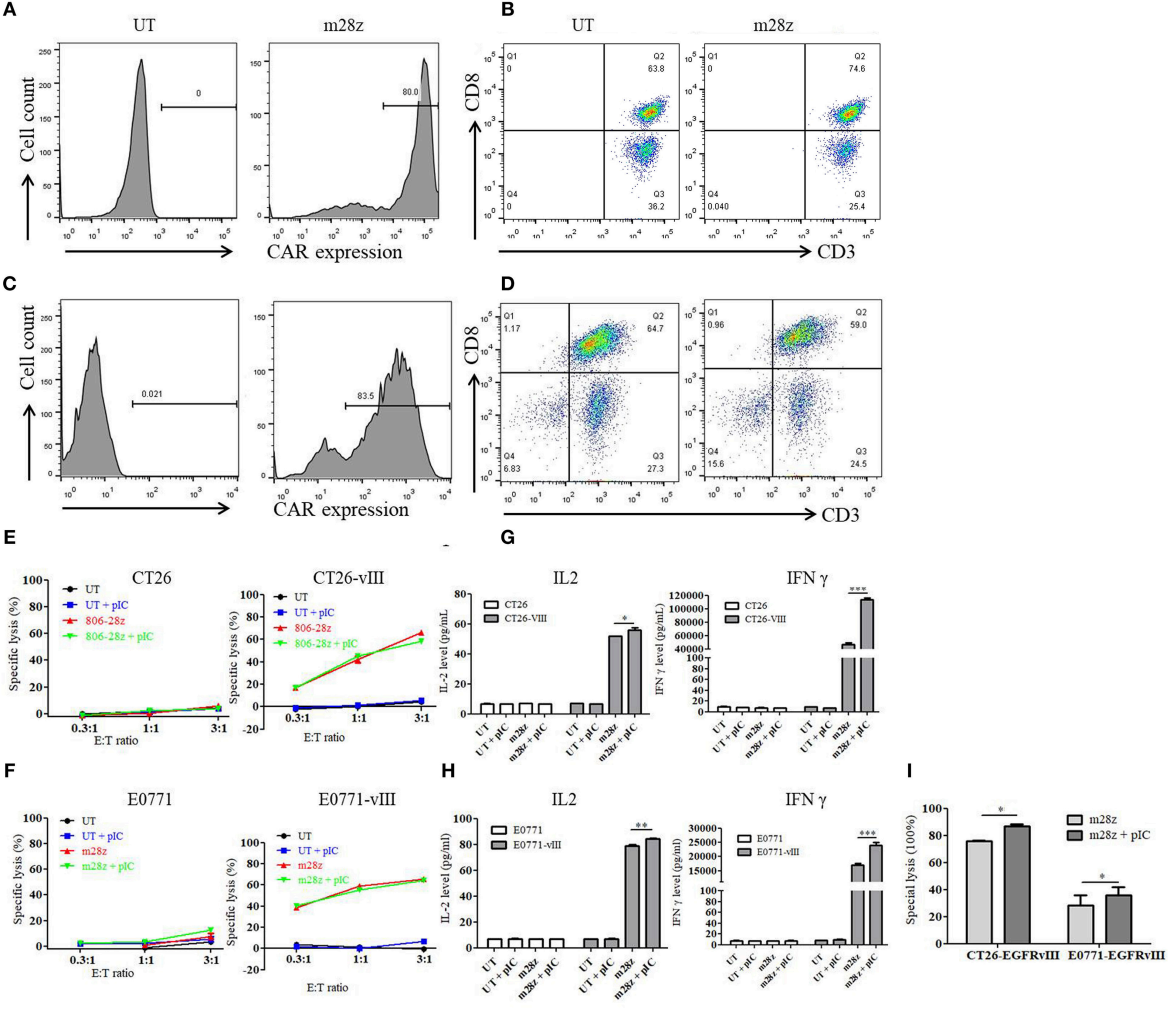
Transduction efficiency, phenotype, and *in vitro* activity of murine CAR-T cells. **(A)** transduction efficiency of CAR-T cells derived from Balb/c mice was detected by flow cytometry. **(B)** three days after transduction, CD3 and CD8 expressions of T cells from **(A)** were analyzed. **(C)** transduction efficiency of CAR-T cells derived from C57 mice was detected by flow cytometry. **(D)** three days after transduction, CD3 and CD8 expressions of T cells from **(B)** were analyzed. **(E)** *in vitro* cytotoxicity of murine CAR-T cells co-cultured with CT26 and CT26-EGFRvIII cells for 18 h was detected with or without poly I:C. **(F)** *in vitro* cytotoxicity of murine CAR-T cells co-cultured with E0771 and E0771-EGFRvIII cells for 18 h was detected with or without poly I:C. **(G)** IL-2 and IFN γ secretion of CAR-T cells co-cultured with CT26 and CT26-EGFRvIII cells was detected by ELISA. **(H)** IL-2 and IFN γ secretion of CAR-T cells co-cultured with E0771 and E0771-EGFRvIII cells was detected by ELISA. **(I)** *in vitro* cytotoxicity of murine CAR-T cells co-cultured with CT26-EGFRvIII or E0771-EGFRvIII cells for 40 h was detected with or without poly I:C. *p < 0.05 **p < 0.01 ***p < 0.001.

We generated murine EGFRvIII specific CAR-T cells by retroviral transduction of splenocytes derived from Balb/c or C57 mice. High transduction efficiency of 80% was obtained as detected by flow cytometry (Figures 1A,B). T cell phenotype was then evaluated, and nearly all cells were CD3 positive. CD8 positive T cells accounted for more than 50% (Figures 1C,D). No significant difference in the percentages of CD3+ or CD8+ cells was found between untransduced T and CAR-T cells.

To determine the impact on *in vitro* activity of CAR-T cells, poly I:C was added to the co-culture of effector cells and target cells. In the cytotoxicity assay, murine CAR-T cells showed significant specific cytolysis activity against CT26-EGFRvIII and E0771-EGFRvIII tumor cells but not CT26 and E0771 cells (Figures 1E,F). However, addition of poly I:C did not enhance the specific activity within 18 h. Additionally, poly I:C neither induced T cell proliferation nor tumor cell death (Supplemental Figure 1).

To further assess the function of poly I:C on CAR-T cells upon antigen-specific stimulation, CAR-T cells were co-cultured with EGFRvIII- negative or positive tumor cells. Supernatant was harvested, and IL-2 and IFN γ levels were measured by ELISA. Upon antigen stimulation for 24 h, we found that CAR-T cells had high level of IL-2 and IFN γ secretion. Moreover, CAR-T cells treated by poly I:C secreted higher level of IL-2 and IFNγ, when compared to the untreated CAR-T cells (Figures 1G,H). However, poly I:C alone could not activate T cells as neither IL-2 nor IFN γ level was increased. Next, we prolonged the co-culture time with an E:T ratio of 0.2:1 for 40 h. As shown in Figure 1I, addition of poly I:C significantly strengthened the *in vitro* killing activity of CAR-T against both CT26-EGFRvIII and E0771-EGFRvIII tumor cells.

### Intratumoral Administration of Poly I:C Enhances *in vivo* Antitumor Activity of CAR-T Cells

**Figure 2:**
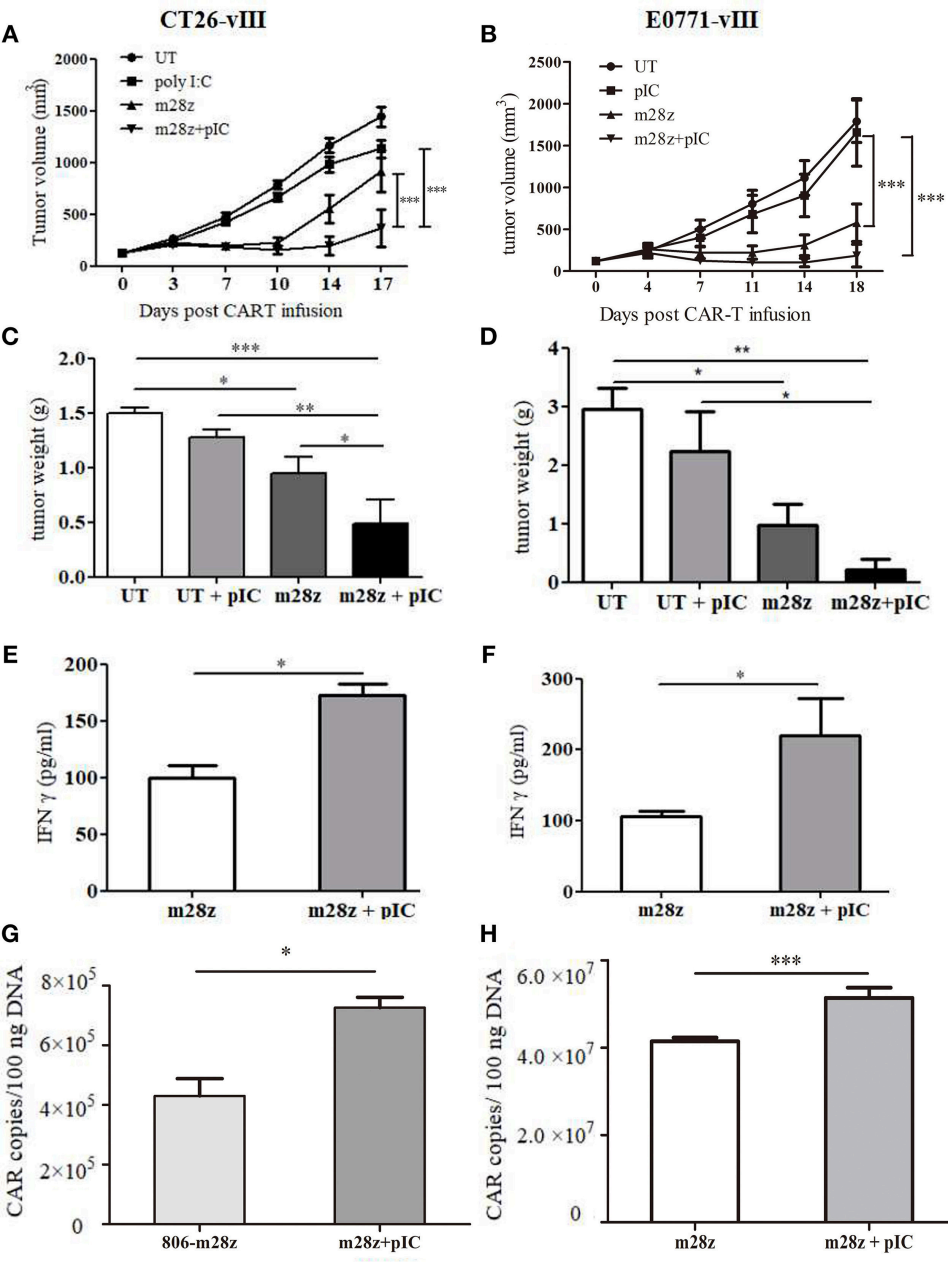
Verification of antitumor effect of poly I:C and CAR-T in murine colon and breast models. Tumor growth in CT26-EGFRvIII **(A)** and E0771- EGFRvIII **(B)** tumor-bearing mice treated by poly I:C and CAR-T cells was monitored; Tumor was weighed at the end of experiment **(C,D)** and IFN γ level in peripheral blood was measured by ELISA **(E,F)**; CAR-T copies in tumor of CT26-EGFRvIII **(G)** and E0771-EGFRvIII **(H)** tumor bearing mice were detected by real-time PCR. *n* = 6/group. *p < 0.05 **p < 0.01 ***p < 0.001.

To address whether poly I:C could increase the antitumor activities of CAR-T cells, mice bearing CT26-EGFRvIII or E0771-EGFRvIII tumor xenografts were treated by EGFR-targeted CAR-T cells, or their combination with poly I:C. Interestingly, we found that intratumoral injections of poly I:C significantly enhance the tumor-suppression activity of CAR-T cells, as showed by the delayed tumor growth (Figures 2A,B) and smaller tumor weight (Figures 2C,D). Additionally, increased amount of IFNγ in the peripheral blood was observed in the group of CAR T cells plus poly I:C (Figures 2E,F). Meanwhile, we also observed that poly I:C alone also mildly suppressed tumor growth. CAR-T persistence is an important factor for successful tumor eradication. At the end of experiment, mice were sacrificed, and genomic DNA was isolated from spleen and tumor to detect CAR copy number by means of quantitative real-time PCR. The results showed that CAR-T poly I:C treatment obviously increased CAR-T copies in tumor tissues (Figures 2G,H).

In addition, to explore whether poly I:C injection caused severe toxicity *in vivo*, body weight of mice was monitored. Results showed that no significant difference in body weight was observed between poly I:C treatment and corresponding control group in both animal models (Supplemental Figure 2).

### Role of Poly I:C-induced IFN β in Poly I:C Mediated Antitumor Responses

**Figure 3:**
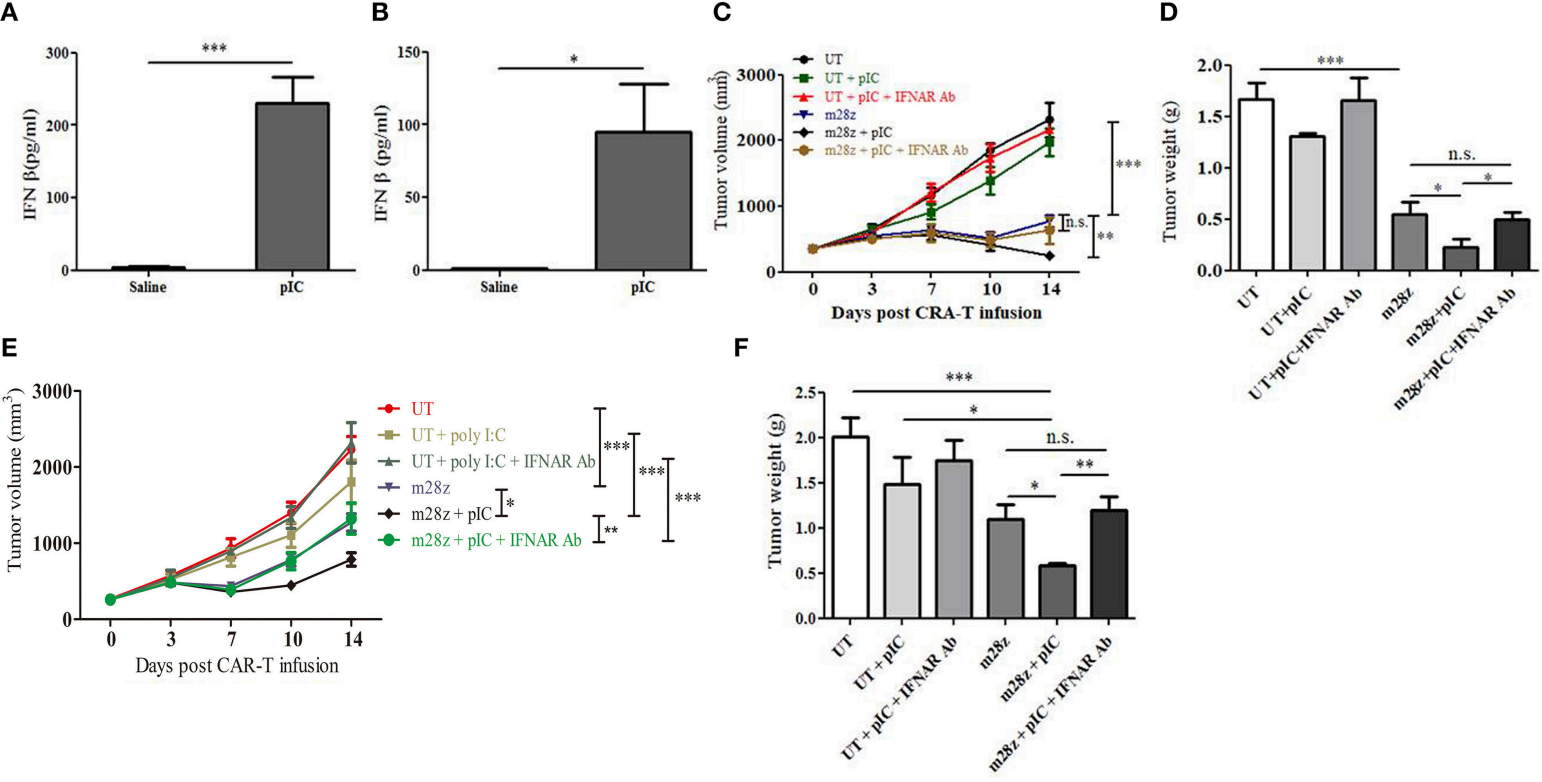
Blockade of IFNAR reverted poly I:C enhanced antitumor activity in the CT26-EGFRvIII and E0771-EGFRvIII tumor-bearing mice. High level of IFN β was induced 3 h post intratumoral delivery of poly I:C in the serum of CT26-EGFRvIII **(A)** and E0771-EGFRvIII **(B)** tumor-bearing mice; Tumor volume was measured in CT26-EGFRvIII tumor-bearing mice **(C)** and tumors were stripped and weighed at the end of experiment **(D)**; Tumor volume **(E)** and tumor weight **(F)** were also obtained from E0771-EGFRvIII tumor-bearing mice treated by poly I:C and CAR-T in the absence or presence of anti-IFNAR Ab. *n* = 6/group. *p < 0.05 **p < 0.01 ***p < 0.001.

Poly I:C is a potent activators of systemic type I IFN responses (24). Upon poly I:C recognition, TLR3 and MDA5 initiate downstream signaling pathways that lead to activation of transcription factors, including IRF3 and NF-κB, which induce type I IFN production (12). In tumors, type I IFNs are associated with increased CTL response (25). In our present study, intratumoral delivery of poly I:C resulted in marked IFN β secretion in peripheral blood of CT26-EGFRvIII (Figure 3A) and E0771-EGFRvIII (Figure 3B) tumor-bearing mice. This induction was temporal, as it was only detectable at 3 h by ELISA but un-detectable 24 h post poly I:C treatment in the serum. To test whether type I IFNs were required for the poly I:C-mediated antitumor effect of CAR-T cells *in vivo*, mice bearing CT26-EGFRvIII or E0771-EGFRvIII tumors were treated with intratumoral injections of IFNAR-blocking Ab on days 0 and 2 after injection with poly I:C or saline (22). Type I IFN signaling blocking impaired the therapeutic effect of poly I:C in CT26-EGFRvIII tumor-bearing mice (Figures 3C,D). Similar trend was also observed in E0771-EGFRvIII tumor- bearing mice treated by poly I:C and CAR-T, when type I IFN signaling was blocked (Figures 3E,F). These results suggested that type I IFNs are essential for the improved antitumor effect of poly I:C and CAR-T cells. To detect whether IFN β could directly enhance CAR-T cell activity, we prepared IFNβ producing CAR-T cells. However, IFNβ was surprisingly unable to enhance cytotoxicity of CAR-T cells against CT26-EGFRvIII and E0771-EGFRvIII when used *in vitro* (Supplemental Figure 3).

### Poly I:C Decreased MDSC Content in Peripheral Blood and Spleen in Tumor-bearing Mice

**Figure 4:**
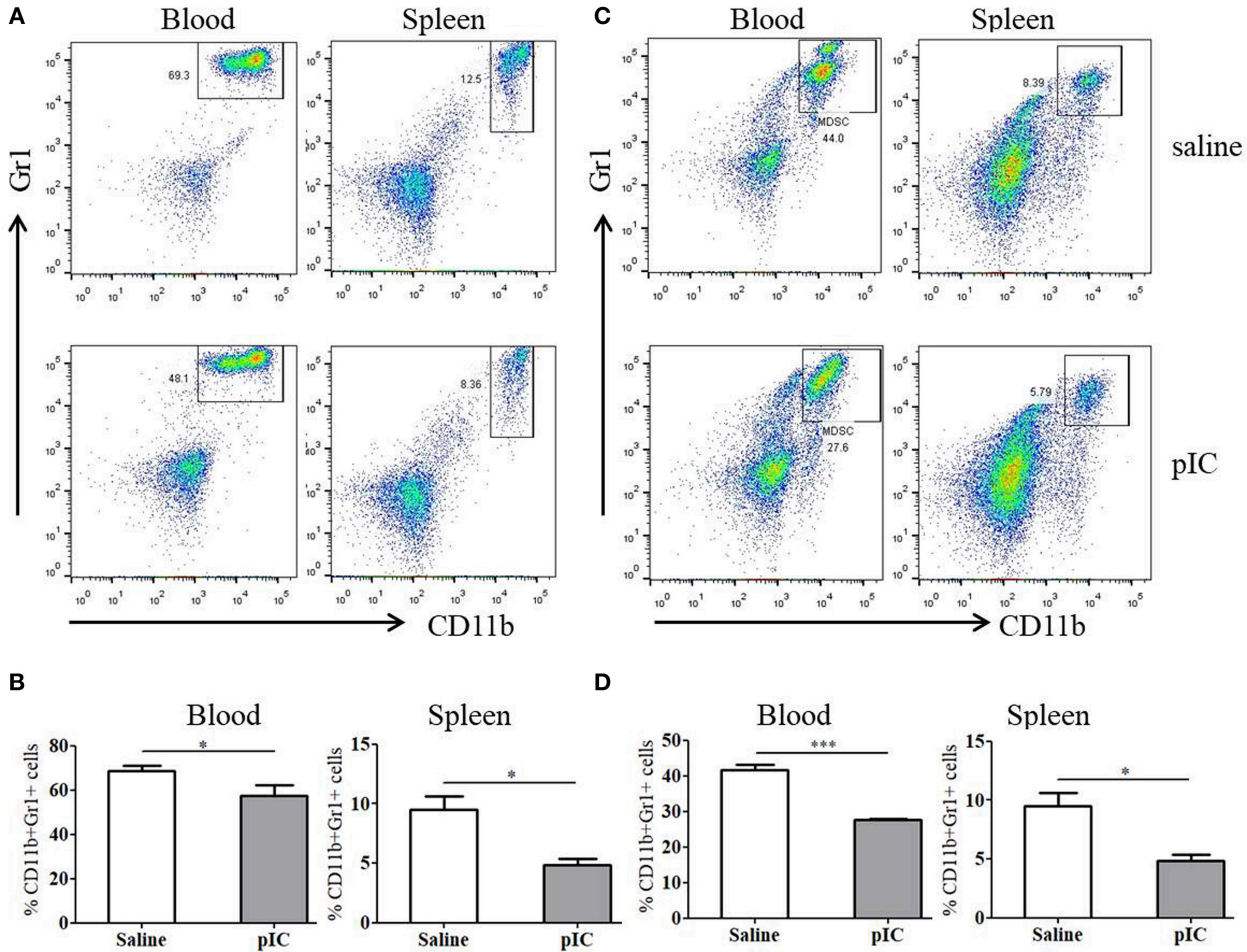
Administration of poly I: C decreases MDSC subunit cell count in tumor-bearing mice compared with saline-treated tumor-bearing mice. **(A)** representative FACS plots gated on CD11b+ Gr1+ cells in peripheral blood, spleen and tumor tissues of CT26-EGFRvIII tumor-bearing mice. **(B)** cumulative data from A, showing the relative ratio of MDSC. **(C)** representative FACS plots gated on CD11b+ Gr1+ cells in peripheral blood, spleen, and tumor tissues of E0771-EGFRvIII tumor-bearing mice. **(D)** cumulative data from B, showing the relative ratio of MDSC. *p < 0.05, ***p < 0.001.

Previous reports showed that tumor growth induces expansion of MDSC in peripheral blood. In turn, these immunosuppressive MDSC contributed to tumor growth (26). Indeed, compared to tumor-free mice, MDSC content was significantly increased in CT26-EGFRvIII tumor-bearing mice (Supplemental Figure 4). To determine whether poly I:C impacts MDSC percentage, tumor-bearing mice were sacrificed 24 h following last poly I:C treatment. MDSC population was analyzed by flow cytometry. As expected, poly I:C treatment significantly decreased MDSC ratio in blood and spleen of mice bearing CT26-EGFRvIII xenograft (Figures 4A,B). However, this difference was not significant in tumor tissues between two groups (Supplemental Figure 5). Similar trends were also observed regarding the number of MDSCs in E0771-EGFRvIII xenograft models treated by poly I:C and CAR-T cells (Figures 4C,D).

### Poly I:C Diminishes Suppressive Function of MDSC in Tumor-bearing Mice

**Figure 5:**
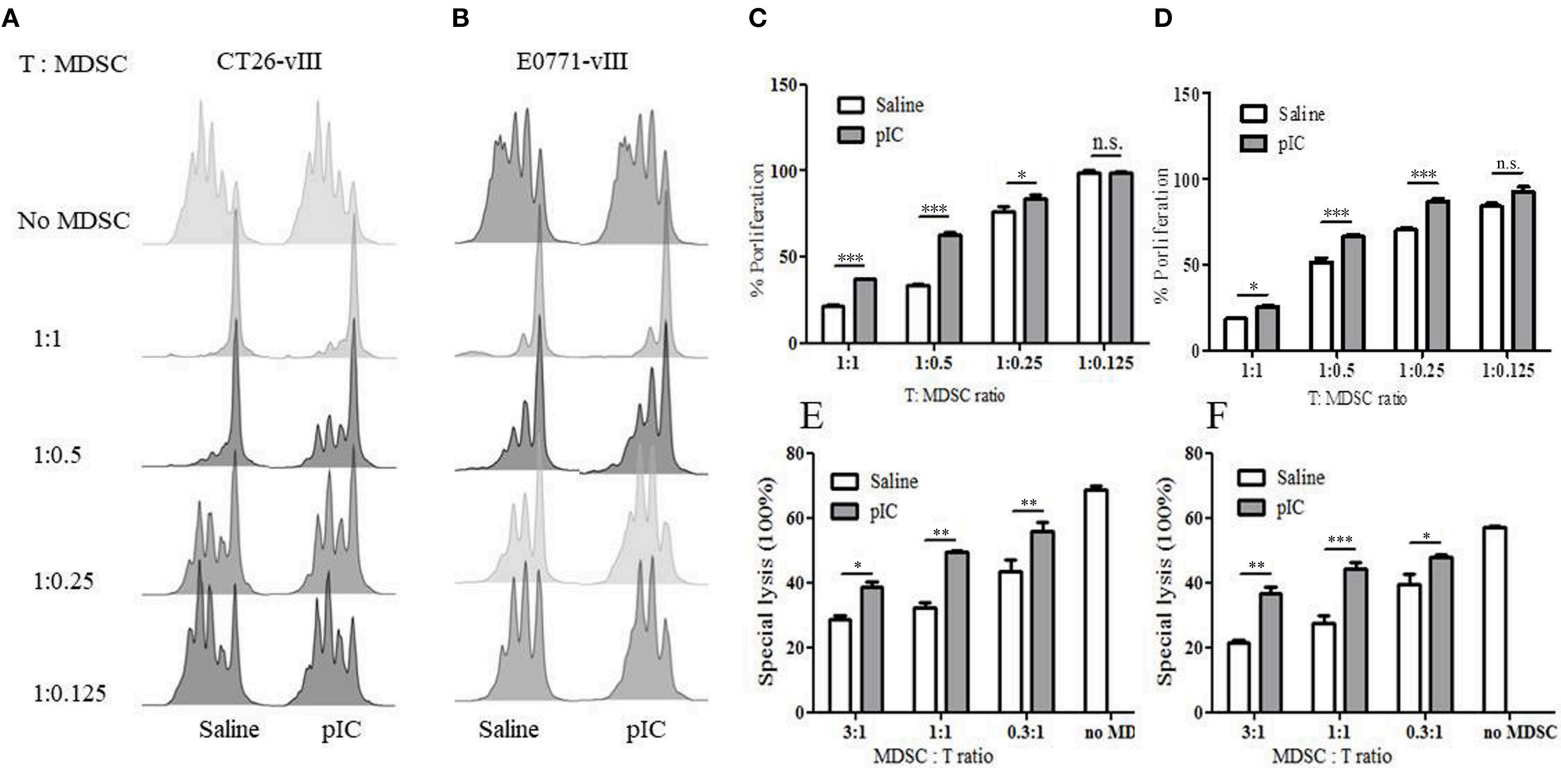
Intratumoral administration of poly I: C attenuated MDSC suppression. **(A)** CFSE dilution of murine T cells following co-incubation with MDSC isolated from saline or poly I:C treated CT26-EGFRvIII tumor-bearing mice. **(B)** representative FACS chart of T cell proliferation co-cultured with MDSC isolated from E0771-EGFRvIII tumor-bearing mice; aCD3/aCD28 beads induced T cell proliferation without MDSC in co-culture was used as positive control. **(C)** proliferation percentage relative to positive control obtained from **(A)**. **(D)** proliferation percentage relative to positive control obtained from **(B)**. **(E)** *in vitro* lytic activity of CAR-T against CT26-EGFRvIII co-cultured with MDSC isolated from CT26-EGFRvIII tumor-bearing mice. **(F)** *in vitro* lytic activity of CAR-T against E0771-EGFRvIII co-cultured with MDSC isolated from E0771-EGFRvIII tumor-bearing mice. Error bars show + SEM. *p < 0.05 **p < 0.01 ***p < 0.001.

To examine whether poly I:C affects the suppressive function of MDSC, we isolated MDSC from the spleen of tumor-bearing mice treated by poly I:C or saline, and co-cultured these cells with splenic T cells of healthy mice. We found that MDSC from poly I:C treated CT26-EGFRvIII and E0771-EGFRvIII tumor-bearing mice were significantly less suppressive on the beads-activated T cell proliferation than that isolated from mice treated with saline, as manifested by CFSE expression (Figures 5A,B) and cell proliferation activity (Figures 5C,D).

To determine whether the decline of MDSC suppression activity following poly I:C treatment could also be observed in cytotoxicity, we examined the specific lytic function of CAR-T cells on target cells with the presence of MDSC. As shown in Figures 5E,F, MDSC inhibited the lytic activity of CAR-T cells in a dose-dependent manner.

These data suggested that MDSCs from poly I:C treated tumor-bearing mice showed diminished suppressive potency compared with those from saline treated mice.

### MDSC Depletion Enhances Efficacy of CAR-T Cells *in vivo*

**Figure 6:**
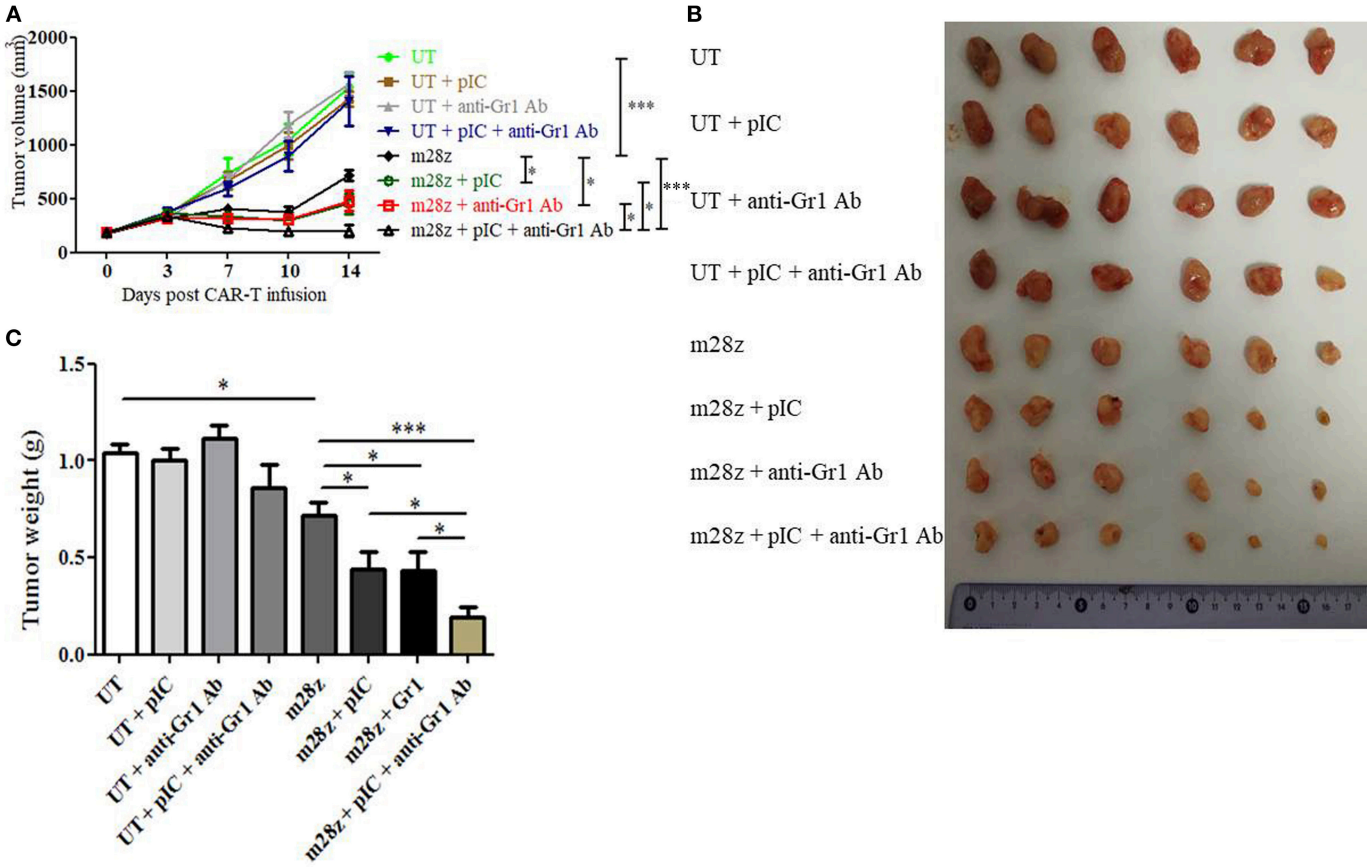
Co-administration of CAR-T with poly I:C and MDSC depletion agent dramatically improved antitumor efficacy against CT26-EGFRvIII subcutaneous tumors. **(A)** tumor growth curve of tumor-bearing mice treated by poly I:C, anti-Gr1 Ab and CAR-T cells. **(B)** At the end of experiment, tumors were isolated from tumor-bearing mice and photographed. **(C)** At the end of experiment, tumors were isolated from tumor-bearing mice and weighed. Error bars show + SEM. *n* = 6/group. *p < 0.05, ***p < 0.001.

As shown above, poly I:C treatment reduced MDSC quantity and diminished MDSC suppression on T cell proliferation and CAR-T lytic activity. Furtherly, we wanted to know whether the enhanced function of poly I:C was mainly due to the inhibition of MDSC. Mice bearing subcutaneous CT26-EGFRvIII tumor were i.p. injected with MDSC depletion agent one day prior to CAR-T cells injection. The depletion of CD11b+ Gr1+ MDSC in peripheral blood was verified by flow cytometry (Supplemental Figure 6). Treatment with MDSC depletion agent alone showed no significant antitumor effect compared to that of UT group (Figure 6). However, treating tumor-bearing mice with both Anti-Gr1 antibody and CAR-T cells led to enhanced antitumor effect, with suppressed tumor growth and less tumor weight, compared with that of mice receiving MDSC depletion agent plus UT cells (Figure 6). This suppression was comparable with that of mice treated by CAR-T combined with poly I:C, as the two groups have almost equal tumor size and tumor weight. Next, we observed that treatment with the combination of CAR-T, poly I:C and MDSC depletion agent (referred to as “tritherapy”) dramatically induced regression of established tumors compared with anti-Gr1 antibody, CAR-T or poly I:C alone.

## Discussion

Immunosuppressive microenvironment has been regarded as the major issue for the limited success of CAR-T therapy on solid tumors. Combination of CAR-T therapy with immune checkpoint molecule inhibitors has been regarded as a promising strategy. For example, a patient with DLBCL and progressive lymphoma was first infused with CAR-modified T cells directed against CD19, and then was administered with anti-PD-1 antibody. Subsequently, the patient had a clinically significant antitumor response, an expansion of CAR-T cells, and decreased coexpression of PD-1 on CAR-T cells (27). Besides, in mice orthotopic glioma models, a single cranial irradiation after tumor implantation further increased the activity of CAR-T cells as demonstrated by synergistically prolonged survival, reduced tumor volume and increased fraction of long-term surviving (28). Here, we aimed to develop a combined immunotherapy that effectively implements CAR-T therapy in solid tumors. Our results supported that poly I:C functioning as an adjuvant aided to enhance CAR-T activity, including specific lysis of target tumor cells and cytokine release upon antigen stimulation. Further studies suggested that poly I:C obviously improved antitumor responses of CAR-T in immune competent mice-bearing colon and breast xenografts.

Systemic administration of poly I:C, including intravenous and intraperitoneal injection, caused significant reduction of body weight and pulmonary thrombosis as well as hepatic necrosis of mice. This toxicity was related to dose and injection schedule. Moreover, weight loss showed a tendency to return to baseline levels with time (29). In the present study, the dose was chosen according to previous publication (13). In this paper, Salmon H et al pointed out that intracellular poly I:C delivery at the tumor site limit potential immune adverse events in other tissues. We also monitored that body weight of mice in experiments and found that poly I:C administration did not cause body weight loss, indicating that local injection of poly I:C did not cause severe systemic toxicity.

To explore the mechanism through which poly I:C enhances CAR-T therapy, we firstly investigated whether poly I:C exert direct impact on tumor and T cells. Previous studies report that poly I:C could directly induce proapoptotic and antiproliferation of many cancer cells (12, 30). However, we did not observe any antiproliferation of poly I:C on both two tumor cells at varying concentrations. Moreover, addition of poly I:C could not directly activate T cells, or promote T cell proliferation even when T cells were simultaneously activated. However, after CAR-T cells were activated by antigen-expressing tumor cells, poly I:C did enhance CAR-T activity, presented by higher levels of IFN γ secretion both *in vitro* and *in vivo*. This was in accordance with previous report that poly I:C cells increased cytokines secretion induced by TCR-dependent and -independent stimulation, but did not affect their proliferation or specific cytolytic activity within short time (31).

One of the main consequences of poly I:C administration is the secretion of type I IFNs. Indeed, we observed obviously elevated IFN β release after poly I:C treatment. Type I IFN signaling pathway blockade almost offsetted the enhanced antitumor activity mediated by poly I:C after CAR-T infusion. Reports demonstrated that type I IFNs can directly and potently induce apoptosis of tumor cells for tumor regression (32). But our study showed that IFN β did not enhance CAR-T cytotoxicity *in vitro*. Thus, we speculated that that IFN β-mediated therapeutic effect requires adaptive immunity, rather than direct killing of tumor cells. This may be due to that IFN β directly targets intratumoral dendritic cells, which reactivate CTL by increasing antigen cross-presentation within the tumor microenvironment (33). In addition, IFN β deficient mice developed faster-growing tumors and these tumors displayed enhanced infiltration by CD11b+Gr1+ neutrophils expressing elevated levels of proangiogenic factors. When treated with low levels of IFN β, however, these tumor-infiltrating neutrophils restored expression of proangiogenic factors to control levels. Moreover, depletion of these neutrophils inhibited tumor growth (34). What's more, type I IFN therapy in mice altered tumor-associated neutrophils polarization toward anti-tumor N1. Similar changes in neutrophil activation could be observed in melanoma patients undergoing type I IFN therapy (35).

MDSCs have emerged as important contributors to solid tumor immune evasion (36). These cells are a heterogeneous population of immature myeloid cells that are expanded by tumors as proved in mouse tumor models and clinical studies. Circulating MDSC numbers correlate with a poor prognosis, tumor vasculogenesis, osteoporosis, and tumor evasion of host immunity (26). They dampen T-cell responses through a number of mechanisms, including depletion of essential nutrients via production of arginase I, iNOS, and indoleamine 2,3 dioxygenase, as well as production of suppressive reactive oxygen species, nitrosylation of chemokines, and expression of PD-L1. So when these cells were depleted by antibody, T cell frequency and function could be restored (26). But in the present CT26-EGFRvIII tumor-bearing mice models, anti-Gr1 Ab injection only slightly delayed tumor growth compared to control.

Tumor-induced MDSC were associated with poor CAR-T cells efficacy against xenografts *in vivo*, implicating the immunosuppressive solid tumor microenvironment as a modulator of CAR-T cell efficacy (36). After poly I:C treatment, the ratio of MDSC in peripheral blood and spleen was significantly decreased. Notably, the intratumoral MDSC percentage was not obviously altered by poly I:C, in which CAR-T cells meet cognate antigen expressing cells. This may be due to that poly I:C induced type I IFN altered tumor-associated neutrophils polarization toward anti-tumor N1 as mentioned above (35).

Poly I:C also inhibited the suppressive activity of MDSC on T cell proliferation and abrogated the immunosuppressive function of MDSC, concomitant with an increase in local T cell response of the immune system in a murine model of breast cancer (37). Our further study revealed that MDSC from tumor-bearing mice directly inhibited CAR-T lytic activity against target cells, while poly I:C treatment significantly decreased this suppression.

In summary, our study for the first time shows that administration of poly I:C can significantly enhance the therapeutic efficacy of CAR T-cell therapy in solid tumors. We also demonstrate the need for mobilizing host immune systems and limiting microenvironmental immunosuppressive activity of solid tumors. The combination of these two modalities is likely to have a significant impact on increasing the effectiveness of immunotherapy against a number of solid tumors, thus provide a new perspective to adoptive immunotherapy of cancer.

## Author Contributions

SD took part in the vector construction, *in vitro* and *in vivo* experiments. MZ and ZP took part in *in vivo* analysis. RS took part in the cell culture. MC helped a lot in the vector construction. HJ and BS helped a lot in the spleen T cell isolation and stimulation. HL took part in the retrovirus production and flow cytometry. ZL is the principle investigator of our research group.

### Conflict of Interest Statement

ZL was employed by company CARsgen Therapeutics and has ownership interests of combined therapy of CAR-T cells with poly I:C. The remaining authors declare that the research was conducted in the absence of any commercial or financial relationships that could be construed as a potential conflict of interest.
