# Supplementary material for: Combined Adjuvant of Poly I:C Improves Antitumor Effects of CAR-T Cells
Source: Front Oncol. 2019 Apr 17;9:241. doi: 10.3389/fonc.2019.00241 (PMC6481273; doi:10.3389/fonc.2019.00241)
Supplement: Supplementary file 1 [file Data_Sheet_1.docx]

Supplemental materials


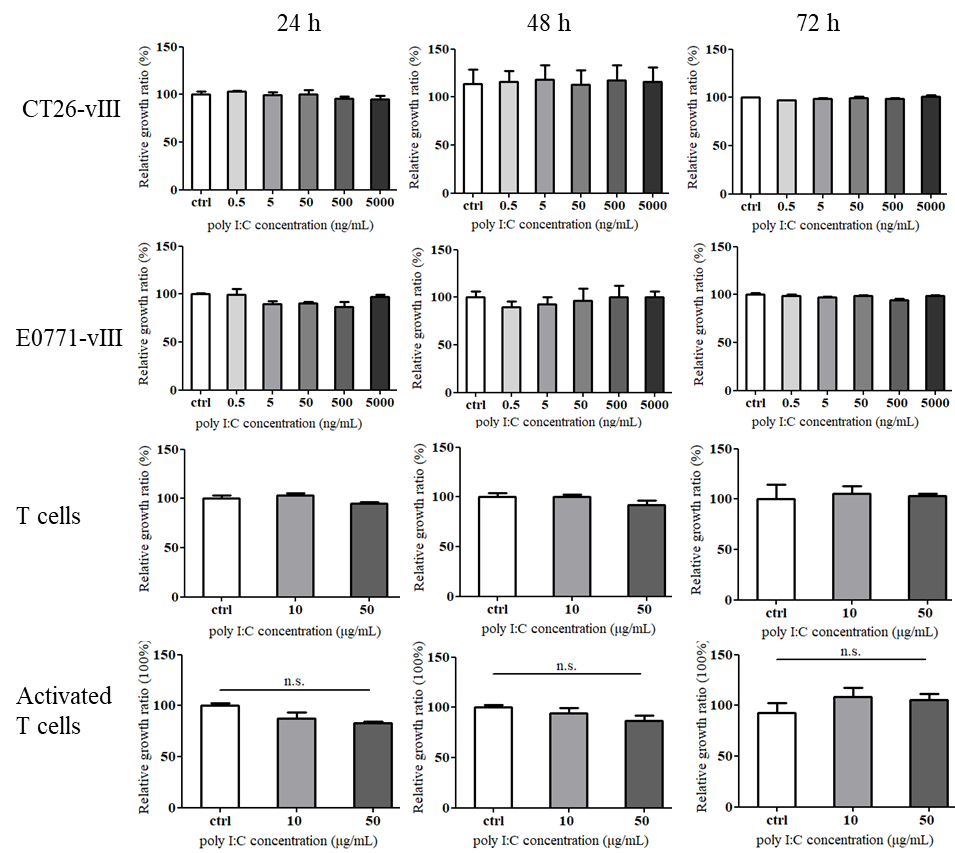


Supplemental Fig 1. Poly I:C did not alter proliferation of tumor cells and T cells. CT26-EGFRvⅢ, E0771-EGFRvⅢ, T and activated T cells were cultured with varying concentrations of poly I:C. After 24 h, 48 h and 72 h, cell proliferation viability was assayed by CCK8 method. All the OD450 nm values of poly I:C treated groups were normalized to control.

Supplemental Fig 2. Body weight was measured in CT26-EGFRvⅢ (left) and E0771- EGFRvⅢ (right) tumor-bearing mice.


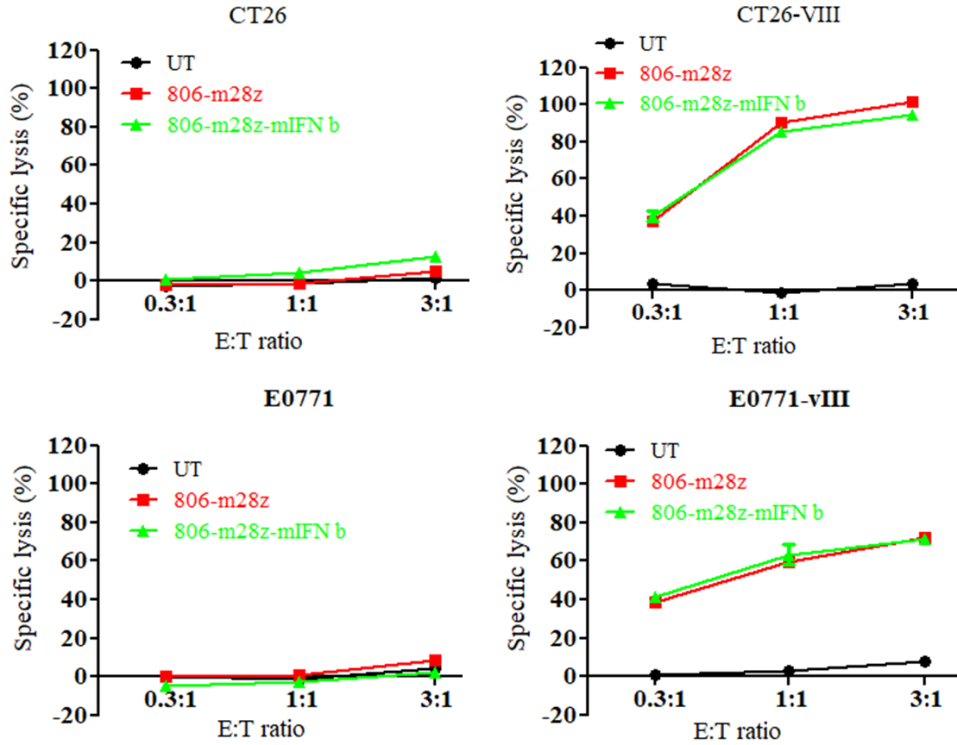


Supplemental Fig 3. In vitro cytotoxicity of IFN β-secreting CAR-T cells against tumor cells.


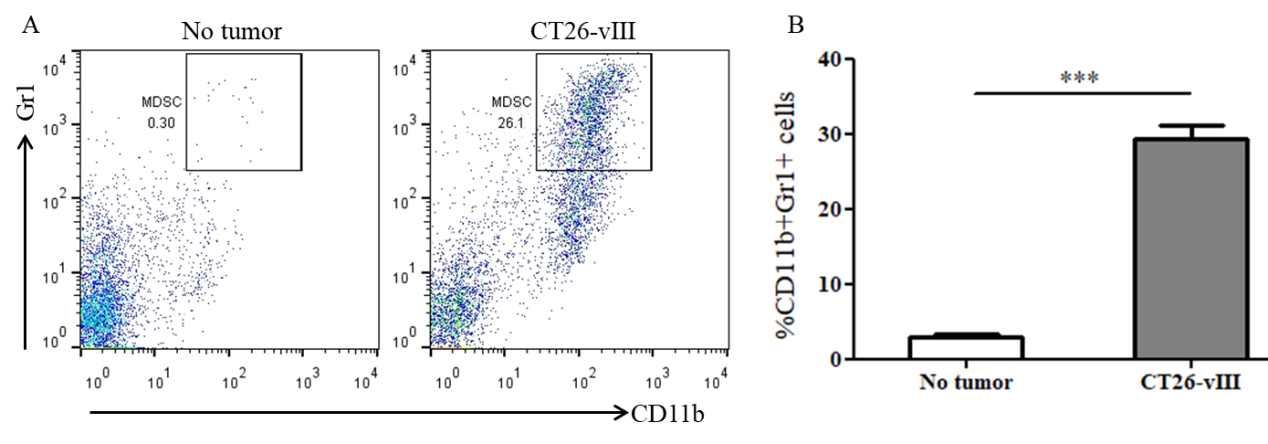


Supplemental Fig 4. MDSC content in spleen of tumor-free and CT26-EGFRvⅢ tumor-bearing mice. **A**: Representative FACS plots gated on CD11b+ Gr1+ cells; **B**: Cumulative data from A, showing the relative ratio of MDSC.


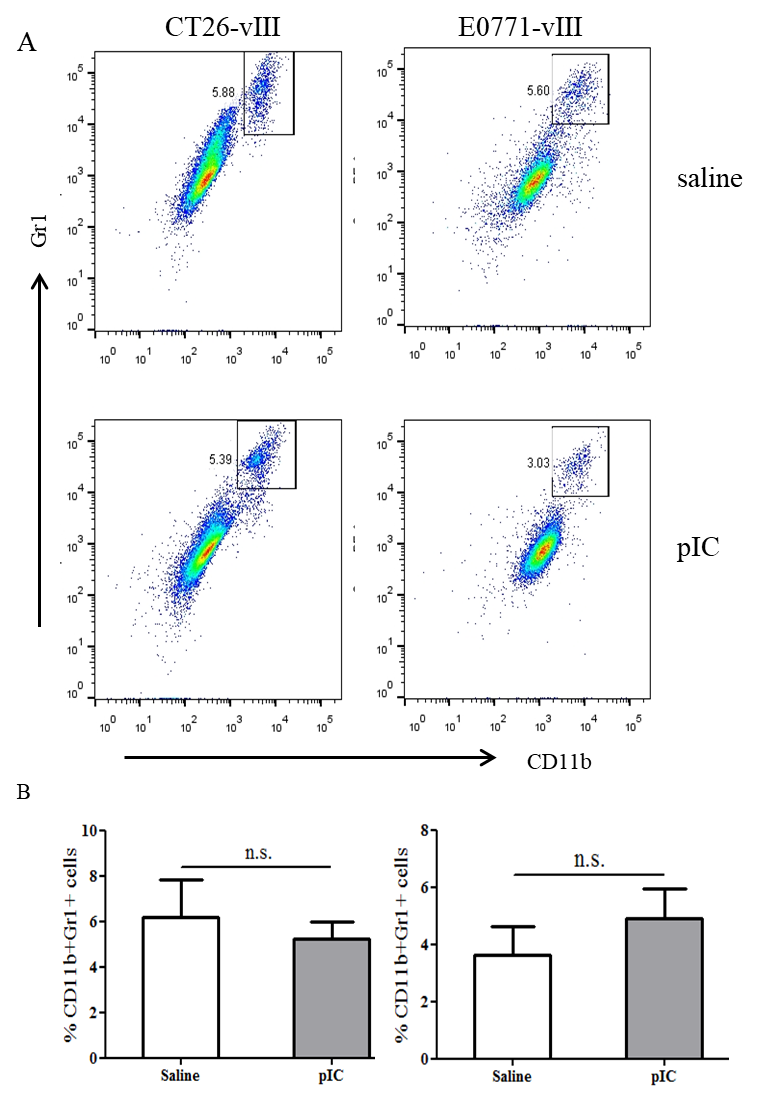


Supplemental Fig 5. MDSC content in tumors of CT26-vⅢ and E0771-vⅢ tumor-bearing mice. **A**: Representative FACS plots gated on CD11b+ Gr1+ cells in tumor tissues; **B**: Cumulative data from A, showing the relative ratio of MDSC.


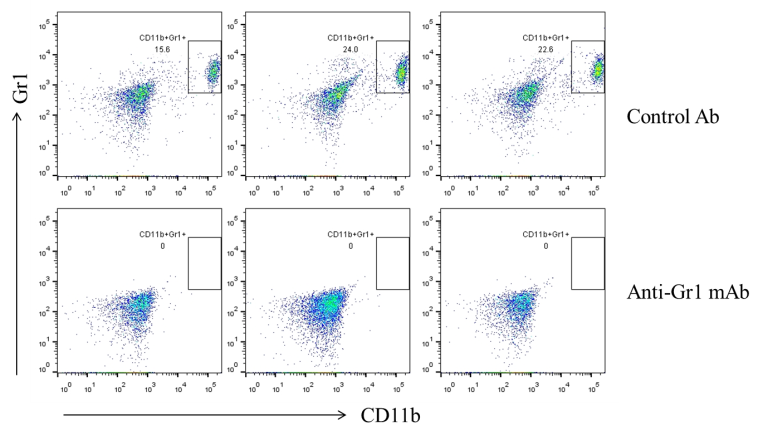


Supplemental Fig 6. Representative FACS plots gated on CD11b+ Gr1+ cells in peripheral blood of CT26-EGFRvⅢ tumor-bearing mice one day later after anti-Gr1 Ab administration.
